# Redox Modulation Matters: Emerging Functions for Glutaredoxins in Plant Development and Stress Responses

**DOI:** 10.3390/plants3040559

**Published:** 2014-11-25

**Authors:** Shutian Li

**Affiliations:** Department of Botany, Osnabrück University, 49076 Osnabrück, Germany; E-Mail: shutian.li@biologie.uni-osnabrueck.de; Tel.: +49-541-969-2272; Fax: +49-541-969-2845

**Keywords:** glutaredoxins (GRXs), glutathione (GSH), thioredoxins (TRXs), redox regulation, development, biotic and abiotic stresses, iron-sulfur clusters

## Abstract

Glutaredoxins (GRXs) are small ubiquitous glutathione (GSH)-dependent oxidoreductases that catalyze the reversible reduction of protein disulfide bridges or protein-GSH mixed disulfide bonds via a dithiol or monothiol mechanism, respectively. Three major classes of GRXs, with the CPYC-type, the CGFS-type or the CC-type active site, have been identified in many plant species. In spite of the well-characterized roles for GRXs in *Escherichia coli*, yeast and humans, the biological functions of plant GRXs have been largely enigmatic. The CPYC-type and CGFS-type GRXs exist in all organisms, from prokaryotes to eukaryotes, whereas the CC-type class has thus far been solely identified in land plants. Only the number of the CC-type GRXs has enlarged dramatically during the evolution of land plants, suggesting their participation in the formation of more complex plants adapted to life on land. A growing body of evidence indicates that plant GRXs are involved in numerous cellular pathways. In this review, emphasis is placed on the recently emerging functions for GRXs in floral organ development and disease resistance. Notably, CC-type GRXs have been recruited to participate in these two seemingly unrelated processes. Besides, the current knowledge of plant GRXs in the assembly and delivery of iron-sulfur clusters, oxidative stress responses and arsenic resistance is also presented. As GRXs require GSH as an electron donor to reduce their target proteins, GSH-related developmental processes, including the control of flowering time and the development of postembryonic roots and shoots, are further discussed. Profiling the thiol redox proteome using high-throughput proteomic approaches and measuring cellular redox changes with fluorescent redox biosensors will help to further unravel the redox-regulated physiological processes in plants.

## 1. Introduction

**Figure 1:**
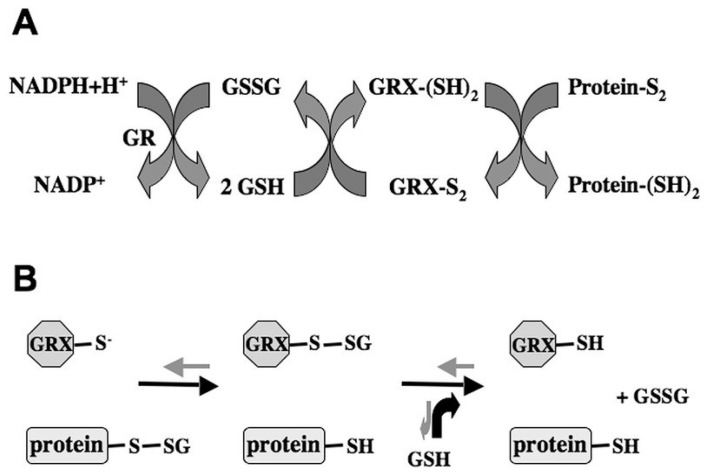
GRXs reduce disulfide bridges via a dithiol (**A**) or monothiol (**B**) mechanism. (**A**) Dithiol mechanism: electrons are transferred from NADPH to glutathione reductase (GR), then to glutathione (GSH) and further to glutaredoxin (GRX), eventually leading to the GRX-mediated reduction of disulfides in target proteins. (**B**) Monothiol mechanism: reduction of protein-GSH mixed disulfides is termed deglutathionylation (black arrows). The reverse reaction is called glutathionylation (gray arrows), resulting in the production of glutathionylated proteins. Adapted from [10].

**Figure 2:**
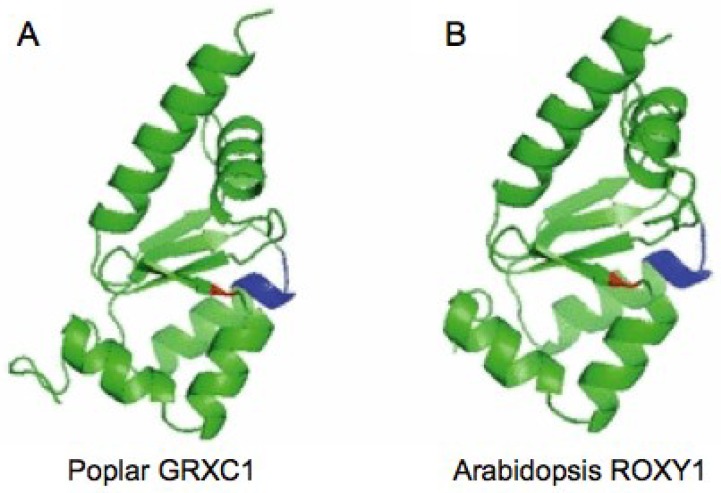
Structural characterization of plant GRXs. (**A**) Ribbon representation of poplar GRXC1. Four central β-sheets are surrounded by five α-helices. The position of the conserved glycine in the GSH binding site is indicated in red, and the CGYC active site is blue. (**B**) Ribbon representation of *Arabidopsis* ROXY1 obtained by homology modeling using poplar GRXC1 as a template. The conserved glycine (G110, red) and the CCMC active site (blue) are respectively located on the same β-sheet and α-helix as in poplar GRXC1. Adapted from [7].

Glutaredoxins (GRXs) are small ubiquitous glutathione (GSH)-dependent oxidoreductases that are widely known to play a crucial role in the response to oxidative stress in *E. coli*, yeast and humans [1,2]. Together with thioredoxins (TRXs), protein disulfide isomerases (PDIs), glutathione-S-transferases (GSTs) and glutathione peroxidases, GRXs are classified into the TRX superfamily sharing a conserved β1-α1-β2-α2-β3-β4-α3 TRX fold [3]. Both TRXs and GRXs belong to a multigenic family of proteins, which are represented by numerous isoforms localized in multiple subcellular compartments [4]. GRXs are able to reversibly reduce disulfide bridges of substrate proteins through two different mechanisms (Figure 1) and thereby posttranslationally influence protein activities, as well as DNA binding and/or transcriptional activities in the case of transcription factors [1,2,5]. In the dithiol mechanism (Figure 1A), GRXs use GSH as electron donors to reduce target proteins through a dithiol-disulfide exchange reaction in a manner similar to that of TRXs. Alternatively, GRXs can also efficiently and specifically catalyze the reversible reduction of protein GSH-mixed disulfides via the monothiol mechanism (Figure 1B), a process known as deglutathionylation [1,6]. In accordance with the predicted amino acid sequences and the composition of the active site motif, GRXs are divided into three major classes, including the CPYC-type, the CGFS-type and the CC-type [6]. Not all of the dithiol GRXs of the CPYC-type contain the exact CPYC active site, as P is replaced by G and S in GRXC1 and GRXC5, respectively [6]. Moreover, the monothiol GRXS12 with the CSYS active motif is phylogenetically more related to GRXC5 than to the CGFS-type GRXs and consequently falls into the CPYC-type [6]. The first structural insight into poplar GRXC1 with a CGYC active site enables us to perform homology modeling for a given GRX using it as a template (Figure 2) [7,8]. Whereas the dithiol CPYC-type and the monothiol CGFS-type are common to all pro- and eukaryotes, the CC-type class is only specific for land plants [4,6,9,10]. Despite the well-recognized roles of plant TRXs in chloroplastic and mitochondrial processes, seed development and germination, as well as self-incompatibility [2], little has been reported to date with respect to the physiological roles of plant GRXs.

The knowledge of plant GRXs has been significantly advanced by the genomic and EST sequence datasets of several plant model species, which uncovers an unexpected number of genes encoding GRXs and, hence, allows for the comparative and evolutionary analysis of GRX classes in aerobic photosynthetic organisms representing different evolutionary stages of plants (Table 1) [4,6,10]. The green alga, *Chlamydomonas reinhardtii*, and the cyanobacterium, *Synechocystis* sp. PCC6803, are unicellular, and both possess the CPYC-type and CGFS-type GRXs, but no CC-type GRXs exist in these primitive photosynthetic organisms (Table 1). The bryophyte *Physcomitrella patens* represents the basal nonvascular land plant and contains only two CC-type GRXs (Table 1). The number of CC-type GRXs expands from the bryophyte to the gymnosperm, *Pinus taeda*, and then to the angiosperms, including the monocot, *Oryza sativa* (rice), as well as the dicots, *Populus trichocarpa* (poplar) and *Arabidopsis thaliana* (Table 1). By contrast, the size of the other two classes remains relatively constant during the land plant evolution, varying from four to six for the CPYC-type and four to eight for the CGFS-type (Table 1). Compositional analysis of three GRX classes in the evolutionarily representative plant species indicates that the exclusive expansion of the land plant-specific CC-type GRXs contributes to plant terrestrial adaptations concomitant with the development of sophisticated and differentiated architectures.

**Table 1:** The respective number of three GRX types in the evolutionarily representative plant species.

Species	CGFS-Type	CPYC-Type	CC-Type
*Chlamydomonas reinhardtii* (green alga)	4	2	0
*Synechocystis* sp.* PCC6803* (cyanobacteria)	1	2	0
*Physcomitrella patens* (moss)	6	4	2
*Pinus taeda* (loblolly pine)	8	4	5
*Oryza sativa* (rice)	5	5	17
*Populus trichocarpa* (black cottonwood)	6	5	22
*Arabidopsis thaliana* (*Arabidopsis*)	4	6	21

CC-type GRXs from *Arabidopsis*, rice and *Zea mays* (maize) have been implicated in floral organ development, defense responses to pathogens and protection against oxidative stress [11,12,13,14,15,16]. This land plant-specific GRX class might have been recruited to participate in these different pathways during the evolution of land plants [12,13,16]. Emerging evidence supports additional roles for plant GRXs in the assembly and transfer of iron-sulfur clusters, oxidative stress responses, as well as arsenic resistance [17,18,19,20]. In addition, GRXs may directly participate in GSH-associated developmental processes, as they require GSH to reduce substrate proteins. Partially inactivating the first enzyme of GSH biosynthesis coupled with knocking out NADPH-dependent TRX reductase genes (*NTRA* and *NTRB*) interferes with auxin homeostasis and leads to the growth inhibition of postembryonic roots and shoots [21,22,23,24,25,26], demonstrating that one of the GRX and TRX systems is required for the postembryonic activities of apical meristems and that the genetic cross-talk between these two redundant systems provides a safety backup to ensure normal postembryonic development. Here, we summarize established roles for plant GRXs in known biological processes with an emphasis on recent advances in developmental processes and defense responses. Furthermore, high-throughput proteomic approaches and fluorescent redox biosensors are also discussed regarding their respective application in profiling the thiol redox proteome and measuring cellular redox changes.

## 2. GRXs with a CGYC or CGFS Active Site Act in the Assembly of Iron-Sulfur Clusters

Iron-sulfur (Fe-S) clusters are ubiquitous in all living organisms and exist in a variety of Fe-S proteins involved in various essential biological processes, such as photosynthesis, metabolism and respiration [27,28,29,30]. In plants, the biogenesis of Fe-S clusters mainly occurs in mitochondria and chloroplasts, utilizing the components of the Fe-S cluster assembly (ISC) machinery and the sulfur mobilization (SUF) machinery as potential scaffold proteins, respectively [31,32]. These two plant organelles contain many Fe-S proteins, such as [2Fe-2S] ferredoxin and [4Fe-4S] ferredoxin-thioredoxin reductase, and are known to participate in the biosynthesis of organellar and cytosolic Fe-S proteins [33]. However, less is currently known about the detailed mechanism controlling the assembly and transfer of Fe-S clusters in plants.

Biochemical and spectroscopic studies have indicated specific roles for plant GRXs with a CGFS or CGYC active site motif in the biosynthesis and delivery of Fe-S clusters [8,17,19,34]. The subunit-bridging [2Fe-2S] cluster present in holo GRXs is ligated by the catalytic cysteines of two GRXs, as well as the cysteines of two external GSH molecules [8,19,35]. GRXC1 and its orthologs are cytosolic CPYC-type GRXs with a CGYC active site and occur exclusively in dicotyledonous plants [8,19,34]. GRXC1 exists predominantly as a holodimeric form, suggestive of the presence of a Fe-S cluster* in vivo* [34]. The reductive activity of *Arabidopsis* GRXC1 is modulated by the redox-dependent stability of the subunit-bridging [2Fe-2S] cluster, suggesting that GRXC1 acts as a redox sensor to reduce downstream signaling steps under oxidative conditions [34]. Cytosolic GRXC2 is the closest paralog of GRXC1, possesses a CPYC active site, occurs in all seed plants and is unable to accommodate a Fe-S cluster [34]. Mutagenesis analysis of poplar GRXC1 indicates that the incorporation of a [2Fe-2S] cluster is likely characteristic of plant GRXs possessing a glycine adjacent to the catalytic cysteine [19]. In support of this notion, the monothiol CGFS-type GRXs of *Arabidopsis* and poplar have the potential to function as scaffold proteins for the assembly and delivery of [2Fe-2S] clusters or as the storage and delivery proteins of [2Fe-2S] clusters to mediate the cluster transfer from the ISC or SUF scaffold proteins to the physiologically relevant acceptor proteins [17,35]. Yeast GRX5 is a mitochondrial CGFS-type GRX, and the *grx5* null mutation leads to the enrichment of free iron, oxidative damage, compromised respiratory growth, as well as the impaired assembly and transfer of Fe-S clusters [33,36]. Among the four CGFS-type GRXs encoded by plant genomes, cytosolic GRXS17 (PICOT) and chloroplastic GRXS14 and GRXS16 can fully rescue the defects of the yeast *grx5* mutant, whereas mitochondrial GRXS15 cannot [17]. This complementation experiment demonstrates the capacity of plant monothiol GRXs for the proper biogenesis of Fe-S proteins. Although the dicotyledon-specific GRXC1 is capable of incorporating a [2Fe-2S] cluster to constitute a holoprotein, it fails to further deliver the [2Fe-2S] cluster to an acceptor protein and, thus, lacks the ability to complement the yeast *grx5* mutant [17,34]. The capacity to transfer a [2Fe-2S] cluster to an acceptor protein seems to be specific and exclusive for monothiol GRXs. Comparative studies of GRXS14 and GRXC1 show that this is likely due to the increased accessibility and lability for Fe-S cofactors in monothiol, but not dithiol GRXs [17]. To further address the specificity of plant monothiol GRXs to the biogenesis of Fe-S clusters, it will be of great value to explore their ability to assemble a variety of [2Fe-2S]-containing holoproteins, as well as to evaluate the possibility of their involvement in the maturation of [3Fe-4S] and [4Fe-4S] clusters.

Clearly, the CPYC-type GRXs with the CGYC active site can encompass a stable [2Fe-2S] cluster of unknown function in a homodimeric fashion, and members of the CGFS-type GRXs can also occur as a haloform that binds a labile Fe-S cofactor [17,34]. However, whether the natural CC-type GRXs possess the capacity to assemble a Fe-S cluster still remains an open question. The lack of biochemical properties for this land plant-specific class is due to the inability to produce soluble recombinant proteins [37]. Engineering CC-type GRXs with two soluble poplar CPYC-type GRXs, including GRXC1 and GRXC4, successfully circumvents this obstacle [37]. Divergent from GRXC1, GRXC4 possesses the CPYC active site and fails to bind a [2Fe-2S] cluster [19,34]. Surprisingly, as observed for wild-type GRXC1, each of the mutagenized GRXs with the artificial CCMC/S active site enables the incorporation of a Fe-S cofactor [37], suggesting the capacity of the natural CC-type GRXs to accommodate a Fe-S cluster.

## 3. Land Plant-Specific CC-Type GRXs in Flower Development and Defense Responses

### 3.1. CC-Type GRXs Are Required for Floral Organ Development and Microspore Formation

Genetic dissection of floral homeotic mutants unveils that the combinatorial action of floral homeotic genes specifies floral organ identity in flowering plants [38]. Nevertheless, floral organ primordia are initiated prior to the activation of floral homeotic genes. Up to now, the knowledge about the genes required for the initiation of floral organ primordia has been scarce. Unexpectedly, *GRXC7/ROXY1*, which encodes a CC-type GRX, is required for petal initiation and organogenesis in *Arabidopsis* [11]. The *roxy1* mutant initiates a reduced number of petal primordia and forms 2.5 petals on average instead of four petals in wild-type flowers. During further petal morphogenesis, developmental defects, such as the lack of blade expansion and abnormal blade bending, are also observed in the *roxy1* mutant [11]. Additional mutants of CC-type GRXs from different plant species have been functionally characterized, shedding light on a novel unexpected role for these GRXs in anther development and microspore formation [7,15,16]. Although the *Arabidopsis* *roxy2* mutant does not exhibit any floral abnormalities, cytological characterization of the *roxy1 roxy2* double mutant flowers reveals a disruption of anther lobe differentiation and microspore formation, indicative of a redundant role of these two closely related CC-type GRXs in the control of *Arabidopsis* male gametogenesis [7]. Similar to the case in dicots, CC-type GRXs are also required for microspore formation in monocots, including rice and maize [15,16]. *microsporeless1* (*mil1*) codes for a rice CC-type GRX that is localized on meiocyte centromeres [16]. The *mil1* mutant disables the meiotic entry of sporogenous cell progenies and is therefore male sterile [16]. Apparently, rice MIL1 participates in an anther-specific meiosis initiation pathway to commence meiotic division. Furthermore, *GRXC7/ROXY1* homologs from rice are isolated and characterized [14]. Although rice flowers have a characteristic morphology distinct from that of *Arabidopsis* flowers, *OsROXY1* and *OsROXY2* exhibit highly dynamic floral expression patterns and are transiently expressed in young floral organs, similar to those of their *Arabidopsis* counterparts [14]. Microscopic reconstruction of maize anther development in the *male sterile converted anther1* (*msca1*) mutant offers evidence underlining a crucial role for a CC-type GRX to function as a determinant of male germline fate in maize [15]. In *msca1* anthers, centrally positioned presumptive archesporial cells fail to proceed, but instead divide longitudinally to differentiate into vascular bundles eventually [15]. However, reductive, but not oxidative, treatment rescues archesporial cell specification and triggers meiotic fate acquisition in *msca1* anthers, demonstrating that a hypoxic and reducing environment suffices to modify unknown targets of the maize CC-type GRX MSCA1 and, thus, causes archesporial cell formation.

Floral CC-type GRXs have been recently revealed as interacting partners of TGA transcription factors [13,16]. The *Arabidopsis* genome encodes 10 TGA proteins, including TGA1–7, PERIANTHIA (PAN), TGA9 and TGA10 [39]. TGA2, TGA5 and TGA6 operate redundantly to regulate systemic acquired resistance (SAR), whereas TGA1, TGA3 and TGA4 participate in basal resistance [40,41,42,43]. By contrast, PAN is involved in the determination of flower organ number in *Arabidopsis*. The *pan* mutant converts tetramerous flowers to pentamerous ones, a feature of ancestral flowering plants [44,45]. All TGA proteins, including PAN, are identified by yeast two-hybrid assays and bimolecular fluorescence complementation (BiFC) experiments as GRXC7/ROXY1-interacting partners [13,46]. Deletion analysis of GRXC7/ROXY1 unravels the importance of the GRXC7/ROXY1 C-terminal L**LL motif for the interactions with TGA proteins and for the GRXC7/ROXY1 function in petal initiation and morphogenesis [13,47,48]. In agreement with their nuclear interactions, intracellular localization studies of GRXC7/ROXY1 combined with complementation experiments of the *roxy1* mutant reveal that a novel nuclear activity of GRXC7/ROXY1 is required for petal development [13]. Mutagenesis studies of all of the PAN cysteines followed by complementation experiments unveil the indispensability of only the cysteine 340 (Cys-340) for the function of PAN in flower development [13]. Notably, Cys-340 is conserved in several other TGA proteins, such as TGA1 and TGA4 [48]. However, direct evidence in support of this conserved cysteine acting as a redox switch has been lacking to date. Genetic analysis of the *roxy1* and *pan* single mutants shows opposing effects of GRXC7/ROXY1 and PAN on petal primordia initiation [13]. In the *roxy1 pan* double mutant, pentamerous flowers develop, indicating that PAN is epistatic to GRXC7/ROXY1 in the regulation of petal primordia initiation [13]. The chimeric repressor gene-silencing technology (CRES-T) successfully uncouples a dual role of TGA factors, namely the negative effect of PAN on petal initiation and the positive effect of additional TGA factors on later petal morphogenesis [13]. Given the opposite effects of GRXC7/ROXY1 and PAN on petal primordial initiation, GRXC7/ROXY1 seems to inhibit PAN activity. Conversely, GRXC7/ROXY1 likely exerts a positive effect on other TGA factors during later petal development, enabling proper petal morphogenesis [13].

*Arabidopsis* plants mutant for both TGA9 and TGA10 are male sterile and display defects in male gametogenesis [46], which is similar to what is observed in the* roxy1 roxy2* double mutant [7]. The male-sterile phenotypes seen in the *tga9 tga10* double mutant demonstrate a redundant role for *TGA9* and *TGA10* in anther development and microspore formation [46]. More specifically, the development of the adaxial and abaxial anther lobes is differentially affected in both the *tga9 tga10* and *roxy1 roxy2* double mutants, with early steps blocked in adaxial lobes and later steps impaired in abaxial lobes [7,46]. In conjunction with the nuclear interactions of TGA9 and TGA10 with GRXC7/ROXY1 and GRXC8/ROXY2 [46], these genetic data indicate that TGA9 and TGA10 act in concert with GRXC7/ROXY1 and GRXC8/ROXY2 to promote anther development. Like *Arabidopsis* CC-type GRXs, the centromere-localized maize MIL1 also interacts with rice TGA proteins [16], implying that MIL1 might also influence the male meiosis initiation through associating with TGA proteins.

Collectively, reduced petal number in the *roxy1* mutant and the male-sterile phenotype of the aforementioned CC-type GRX mutants suggest that redox regulation plays a significant role in floral organ development and in the production of male germline cells. Nuclear interactions between floral CC-type GRXs and floral TGA proteins further advance our knowledge as to how some TGA proteins contribute to flower development. Flowering plants seem to have evolved an anther-specific mechanism, whereby CC-type GRXs dictate meiotic entry and male gametogenesis via modulating redox homeostasis.

### 3.2. CC-Type GRXs Participate in Disease Resistance

Plant pathogens generally fall into two categories: biotrophs and necrotrophs. To fend off these attackers that differ in lifestyles and infection strategies, plants have evolved complex defense mechanisms. The R protein-mediated hypersensitive response (HR) and salicylic acid (SA)-mediated basal resistance play a crucial role in defense against biotrophs. whereas jasmonic acid (JA) activates a set of *pathogenesis-related* (*PR*) genes distinct from those induced by SA and exerts basal resistance against necrotrophs [49,50]. Nevertheless, the SA- or JA-dependent signaling pathways are not always activated exclusively in response to biotrophs or necrotrophs [51]. When pathogen infection simultaneously induces the synthesis of both SA and JA, plants are able to take advantage of the cross-talk between SA- and JA-mediated signaling pathways to optimize the defense against a single attacker [51]. Furthermore, the cross-communication between SA- and JA-dependent defense pathways also occurs in the defense against multiple pathogens with distinct lifestyles [52]. Several key regulators, such as NON-EXPRESSOR OF PR GENES1 (NPR1), MAP kinase 4 (MPK4), WRKY transcription factors and TGA proteins, are demonstrated to be involved in the cross-talk between SA and JA signaling pathways [53,54].

In recent years, the *Arabidopsis* CC-type GRXs, GRXC9/ROXY19 and *GRXS13/ROXY18*, have been shown to act as novel players implicated in the cross-talk between SA and JA signaling pathways [12,55]. Transcriptional activation of *GRXC9/ROXY19* expression by SA requires TGA proteins, but is partially dependent on NPR1 [12,56]. Moreover, constitutive expression of *GRXC9/ROXY19* impairs JA/ethylene (ET)-induced *PDF1.2* transcription, but fails to affect the expression of *LOX2* and *VSP*, two marker genes of the JA response pathway, implying that this CC-type GRX is involved in the SA-induced suppression of the JA/ET signaling pathways [12]. It is well known that the APETALA2/ETHYLENE RESPONSE FACTOR transcription factor ORA59 acts as the global regulator of JA/ET-induced defense responses [57]. *ORA59* transcription is stimulated by JA or ET, but is contrarily repressed by SA [58,59]. Activation of the *ORA59* promoter by the transcription factor ETHYLENE INSENSITIVE3 (EIN3) is suppressed by coexpressing *GRXC9/ROXY19*. Besides, ET-induced *ORA59* expression is also compromised in transgenic plants ectopically expressing *GRXC9/ROXY19* [60]. Together, these observations highlight the SA-JA/ET antagonism mediated by SA-induced expression of *GRXC9/ROXY19*.

*GRXS13/ROXY18* encodes a second disease resistance-related CC-type GRX, which is phylogenetically closely related to *GRXC9/ROXY19* and participates in defense signaling via functioning as a disease susceptibility gene [55,56]. *GRXS13/ROXY18* transcription is stimulated by the virulent strain of the plant necrotrophic pathogen, *Botrytis cinerea*. Moreover, expression of *GRXS13/ROXY18* is SA-inducible, but is conversely suppressed by JA [55]. After *B. cinerea* infection, mRNA levels of *GRXS13/ROXY18* are significantly increased in JA-related mutants, but are strongly reduced in SA-related mutants [55], demonstrating that SA and JA signaling pathways regulate *GRXS13/ROXY18* expression in opposing ways. Whereas the loss-of-function mutation in *GRXS13/ROXY18* causes enhanced resistance to *B. cinerea*, overexpression of this GRX gene in wild-type plants results in augmented susceptibility to this fungus, lending support to the notion that *GRXS13/ROXY18* is required for the successful colonization of *Arabidopsis* plants by *B. cinerea*.

Interestingly, ectopic expression of *GRXC7/ROXY1*, as well as its rice homologs, *OsROXY1* and *OsROXY2*, in *Arabidopsis* leads to increased accumulation of hydrogen peroxide (H_2_O_2_) and renders transgenic plants highly susceptible to infection by *B. cinerea* [14]. In addition, OsWRKY13 operates not only as an activator of the SA-dependent pathway, but also as a suppressor of the JA-dependent pathway [61]. Overexpression of *OsWRKY13* induces the expression of two rice CC-type GRX genes and enhances rice resistance to *Xanthomonas oryzae* pv. *oryzae* (*Xoo*) and *Magnaporthe grisea*, hinting at the possible involvement of these two CC-type GRXs in the SA-JA antagonism [61,62].

SA-induced GRXC9/ROXY19 interacts with various TGA proteins and negatively regulates JA-responsive *PDF1.2* transcription [12]. The suppressive effect of GRXC9/ROXY19 on *PDF1.2* transcription is abolished in the *tga2 tga5 tga6* triple mutants, indicating that the interaction between GRXC9/ROXY19 and TGA proteins is indispensable for GRXC9/ROXY19-dependent cross-talk between SA and JA signaling pathways [12]. Furthermore, ectopically expressed *GRXC9/ROXY19* suppresses ET-induced expression of *ORA59*, and this repression depends on the C-terminal L ** LL motif of GRXC9/ROXY19 [60]. This short conserved motif was previously found to be essential for the interaction of floral GRXC7/ROXY1 with TGA proteins [47]. Interestingly, 10 of the 21 CC-type GRXs in *Arabidopsis* interact with TGA proteins in yeast two-hybrid assays and repress the *ORA59* promoter activity [60].

Functional analysis of *GRX13/ROXY18*, which encodes the closest paralog of GRXC9/ROXY19, indicates a role for this CC-type GRX gene in facilitating pathogen infection [55]. In wild-type plants, expression of *GRXS13/ROXY18* is undetectable, but is strongly inducible by SA. Nonetheless, the *tga2 tga5 tga6* triple mutation results in slightly elevated expression of *GRXS13/ROXY18* and a severe reduction of SA-induced transcription of *GRXS13/ROXY18* [55,56], suggesting that SA-induced expression of *GRXS13/ROXY18* requires the subclass II of TGA transcription factors (TGA2, TGA5 and TGA6). These TGA proteins are identified as redundant modulators of defense responses in *Arabidopsis* plants challenged with *B. cinerea* [63]. Yeast two-hybrid assays further reveals the capacity of GRXS13/ROXY18 to associate with TGA proteins [55], implying that GRXS13/ROXY18 act together with these TGA factors to trigger a cascade of events leading to disease susceptibility.

Taken together, land plant-specific CC-type GRXs participate in defense responses by cooperating with stress-related TGA proteins. The specific recruitment of CC-type GRXs in plant defense signaling suggests that these small proteins act to maintain cellular redox homeostasis after pathogen infection, which is essential for plant adaptations to life on land under adverse environmental conditions during the evolution of land plants.

### 3.3. Functional Redundancy of a Subset of CC-Type GRXs with a Conserved C-Terminal Motif

Yeast two-hybrid assays and BiFC experiments reveal that both stress-related and floral TGA proteins interact with CC-type GRXs [12,13,16,46,55]. Complementation experiments of the *roxy1* mutant unveil the functional conservation of complementing CC-type GRXs and the functional importance of their C-terminal A [L/I]WL motif in flower organ development [13]. One of these rescuing CC-type GRXs is encoded by *GRXC11*/*ROXY4* (At3g62950), which is upregulated by DELLA proteins and participates in the gibberellin (GA) signaling pathway and floral organ development [64]. Unexpectedly, GRXC9/ROXY19 is a disease resistance-related CC-type GRX, possesses this C-terminal short motif and is able to replace GRXC7/ROXY1 if properly expressed [12,13]. Besides, OsROXY1 and OsROXY2 share the C-terminal ALWL motif and can fully rescue the *roxy1* mutant [14]. The exchangeability between CC-type GRXs is also corroborated by a study focused on the inhibitory effects of both GRXC9/ROXY19 and its homologs on *ORA59* transcription [60]. These suppressive CC-type GRXs have a common C-terminal ALWL motif and are capable of interacting with TGA proteins [60]. Thus, nucleotide changes in the regulatory, but not coding, regions of these CC-type GRX genes contribute to their sub- and neo-functionalization [65]. In sum, these observations demonstrate a conserved function for a subgroup of CC-type GRXs in flower development and disease resistance,* i.e.*, effectively recognizing TGA proteins involved in either of the two signaling pathways. Since CC-type GRXs are required for microspore production and defense response, they can potentially be utilized to engineer disease-resistant crops and to develop male sterile lines for hybrid seed production.

## 4. GRXs Participate in Abiotic Stress Responses

**Figure 3:**
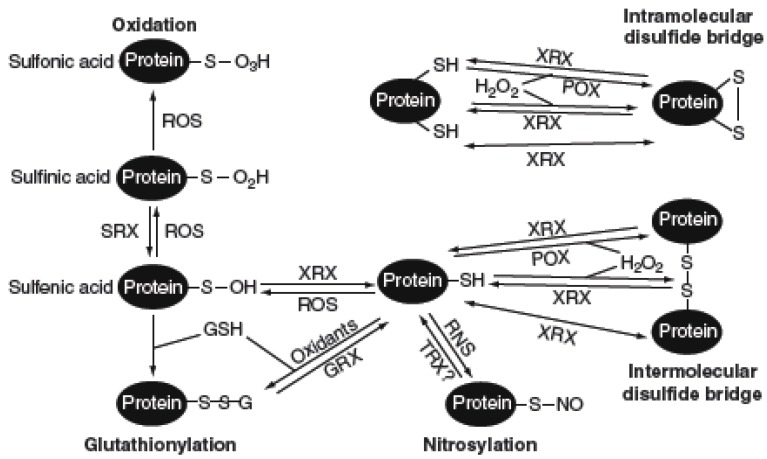
Oxidation and reduction of protein thiols. Under oxidative conditions, free and accessible protein thiols undergo several different posttranslational modifications, which can be either reversible or not. Protein cysteines can be oxidized by ROS into sulfenic acid (SOH), which can be reduced by XRX (GRX or TRX). Further oxidation of sulfenic acid by ROS can result in the formation of sulfinic acid (SO_2_H), which can be reversed by sulfiredoxins (SRX) or irreversibly oxidized to sulfonic acid (SO_3_H). The presence of oxidants and/or GSH allows the glutathionylation of protein cysteines to occur through different mechanisms. Deglutathionylation can be catalyzed by GRX. Reversible formation of intra-/inter-molecular disulfide bridges is mediated by XRX. Direct H_2_O_2_-dependent oxidation of cysteines to intra-/intermolecular disulfides and peroxidase (POX)-catalyzed H_2_O_2_ sensing can be reversed by XRX. Besides, protein cysteines also undergo nitrosylation in the presence of reactive nitrogen species (RNS), a reversible process that could be catalyzed by TRX. Adapted from [48].

In a continuously changing environment, plants are constantly challenged by a plethora of abiotic stresses, such as heavy metals, extreme temperatures, high light and toxic substances. A common characteristic of plant responses to such externally environmental stimuli is an oxidative burst of reactive oxygen species (ROS), which alters cellular redox homeostasis and produces oxidative stress. To overcome such oxidative stress, plants have orchestrated a sophisticated antioxidant mechanism to counteract the deleterious effects of ROS and avoid cellular oxidation [66]. Redox signaling involves posttranslational modifications of protein thiols that can be oxidized into different reversible states, such as *S*-glutathionylation, *S*-nitrosylation and intra-/intermolecular disulfide bridges (Figure 3). These oxidized forms of cysteine residues can be effectively reduced by GRXs, indicating a potential key role for GRXs in oxidative stress signaling (Figure 3) [66,67].

Plants adapt themselves to oxidative stress by coordinately regulating a battery of antioxidant genes. In *Arabidopsis*, at least 152 genes are possibly involved in managing the level of ROS, and 27 of them encode GRXs [68]. Reverse genetic analysis combined with transgenic approaches has identified some *Arabidopsis* GRXs as critical players in protection against oxidative stress [18,34,69,70,71]. Except for GRXS16, all of the other three monothiol GRXs, namely GRXS14 (also known as GRXcp), GRXS15 and GRXS17 (PICOT), seem to exert such antioxidant functions [18,69,70]. Analysis of the *grxs14* mutant demonstrates developmental defects in early seedling growth under oxidative stress [18]. Besides, augmented protein carbonylation is detected within *grxs14* chloroplasts [18]. Despite a ubiquitous expression pattern, transcription levels of *GRXS15* are affected by various abiotic stresses. Genetic experiments reveal that *grxs15* are sensitive to oxidants, implying important functions for GRXS15 in plant growth and development under extreme conditions [69]. The *grxs17* mutant is hypersensitive to high temperature, and the expression of *GRXS17* is induced by elevated temperature [70]. Unexpectedly, auxin sensitivity and polar auxin transport are perturbed in this mutant. Under high temperature, *grxs17* plants accumulate high levels of ROS and exhibit phenotypic characteristics reflecting defects in the cell cycle control [70]. Ectopic expression of *GRXS17* in tomato minimizes chlorophyll photooxidation and lessens the oxidative damage of the cell membrane system under heat stress [72]. These observations firmly establish a mechanistic link between redox homeostasis, auxin signaling and temperature-dependent postembryonic development. Knocking down the CGFS-type GRX gene *SlGRX1* using virus-induced gene silencing increases the sensitivity of tomato plants to the oxidative and salt stresses and reduces drought tolerance, whereas overexpression of *SlGRX1* in *Arabidopsis* significantly improves plant tolerance to the oxidative, drought and salt stresses [73]. Arsenate-activated *Pteris vittata* *GRX5* encodes a CGFS-type GRX and represents an ortholog of *Arabidopsis* GRXS14 [20]. Compared with the arsenic-sensitive fern *P. ensiformis,* *P. vittata* is able to hyperaccumulate arsenic in fronds and displays significantly elevated tolerance to oxidative stress [74]. Expression of *PvGRX5* in *E. coli* mutants suggests that PvGRX5 depends on aquaglyceroporins to play a role in cellular arsenic resistance [20]. In most organisms, aquaglyceroporins act as arsenite channels to move arsenite into and out of cells [75]. Therefore, it is most likely that PvGRX5 regulates intracellular arsenite levels via modulating aquaglyceroporins. Ectopic expression of *PvGRX5* in *Arabidopsis* decreases arsenic accumulation, reduces oxidative damage to intracellular proteins and improves tolerance to arsenic and high temperature [76,77]. Apart from facilitating *B. cinerea* infection of *Arabidopsis* plants as described above [55], GRXS13/ROXY18 with the CCLG active site motif also protects plants from photooxidative stresses [71]. Treatment of *Arabidopsis* seedlings with Mev and high light (HL) enhances *GRXS13/ROXY18* transcription and engenders photooxidative stress responses characterized by heightened superoxide levels [71]. Knocking down *GRXS13/ROXY18* leads to the elevated production of superoxide radicals, inhibits plant growth and lowers plant tolerance to Mev and HL. In agreement, overexpression of this GRX gene enhances protection against the Mev- and HL-induced damage [71], suggesting that GRXS13/ROXY18 limits ROS accumulation and protects plants from damage caused by photooxidative stresses. Divergent from GRXC2 found in all seed plants, the dicotyledon-specific GRXC1 is able to assemble a Fe-S cluster [34]. Under various environmental stresses, the *Arabidopsis* *grxc1* or *grxc2* single mutant shows a wild-type phenotype, yet the *grxc1* *grxc2* double mutant displays a lethal phenotype at early steps after pollination, implying a redundant and vital function shared by GRXC1 and GRXC2 [34].

In response to oxidative stress, GRXs limit ROS production, participate in redox signaling and play an antioxidant role, thereby protecting plants from the cellular oxidative damage. This conserved function might be performed by either direct ROS scavenging or redox regulation of target proteins via their disulfide oxidoreductase or deglutathionylation activities. Alternatively, GRXs might indirectly mediate this function through protein interactions or transcriptional control of gene expression. Despite the demonstrated involvement of CC-type GRXs in the control of ROS levels, it should be noted that the oxidoreductase and deglutathionylation activities of this land plant-specific class have not been characterized so far. Therefore, further biochemical experiments are needed to ascertain the enzymatic properties of CC-type GRXs.

## 5. GRXs Cross-Talk with TRXs in Plant Development and Stress Responses

Proteomic approaches have identified numerous common targets of GRXs and TRXs, supporting the cross-talk between these two plant antioxidant systems [78]. GRXs mainly differ from TRXs by the fact that they use GSH as an electron donor instead of TRX reductases [6]. Plants have an unusually complex complement of TRXs, which consists of six well-defined types (f, m, x, y, h, o and z) and resides in different cellular compartments*,* including the cytosol, the nucleus, mitochondria and chloroplasts [66,79,80]. Typically, chloroplastic TRXs are reduced in light by ferredoxin-dependent heterodimeric TRX reductases (FTR) [81]. By contrast, the reduction of plant cytosolic and mitochondrial TRXs is catalyzed by NADPH-dependent TRX reductases (NTR) [6]. In spite of the divergent reduction modes exerted by these two redox systems, accumulating evidence suggests that GRXs interplay with TRXs to constitute a complex network of redox signaling [25,26,82]. Besides, as oxidized GRXs are recycled by GSH for both the monothiol and dithiol mechanisms, it is likely that GRXs are directly involved in GSH-associated biological pathways.

In plants, GSH is enzymatically synthesized from its constituent amino acids via two ATP-dependent steps, which are catalyzed by γ-glutamylcysteine synthase (γ-GCS) and GSH synthetase (GS), respectively. In *Arabidopsis*, both γ-GCS and GS are encoded by a unique gene [21]. Complete inactivation of *Arabidopsis* *GSH1*, which encodes γ-GCS, causes the formation of white, embryo-lethal seeds [21,83]. Artificially changing endogenous GSH levels coupled with phenotypic analysis of *GSH1* mutants, including *root meristemless1* (*rml1*), *cadmium sensitive2* (*cad2*) and *phytoalexin-deficient2-1* (*pad2-1*), unravels that GSH participates in developmental processes and plant responses to biotic and abiotic stresses, such as heavy metals, ultraviolet (UV)-induced ROS, drought, chilling and pathogens [22,23,24,25,26,84,85,86,87,88,89]. The strong mutant allele *rml1* encodes a very inefficient γ-GCS and reduces the GSH synthesis by 95%, resulting in sterile plants with an extremely short mature root that comprises the same number of cells as the embryonic root [22,88]. This arrest with respect to root growth demonstrates that the *rml1* mutation disables postembryonic cell division in the root. By contrast, cell division still occurs in the apical shoot meristem of the *rml1* mutant, albeit generating a small shoot with leaves, flowers and seeds containing abnormal embryos [88]. Comparatively, the two weak mutant alleles, *cad2* and *pad2-1*, lead to a decline of 80% and 84% in GSH levels, respectively. Except that *cad2* displays a late flowering phenotype [23,24], both the *cad2* and *pad2-1* single mutants are developmentally indistinguishable from wild-type plants [21,89]. In addition, *pad2-1* is sensitive to several pathogens, whereas *cad2* is hypersensitive to cadmium [84,89].

The key reductants of cytosolic and mitochondrial TRXs are NTRs encoded by *NTRA* and *NTRB*, both of which code for cytosolic and mitochondrial isoforms in *Arabidopsis* [90]. Knocking out *NTRA* or *NTRB* fails to reveal any discernible mutant phenotype, indicative of a functional redundancy between them. Although the inactivation of both *NTRs* retards plant growth, changes seed shape, compromises pollen fitness and hyperaccumulates anthocyanins, the *ntra ntrb* double mutant is fully fertile and does not display a significantly altered sensitivity to biotic and abiotic oxidative stresses [25,82]. Moreover, cytosolic TRXh3 is only partially oxidized in the double mutant, implying an alternative mechanism for reducing TRXs [25]. Crossing *ntra ntrb* to the wild-type-like cytosolic GSH reductase mutant *gr1* generates the *ntra ntrb grl* plants with lethal pollen [82]. Associating *ntra ntrb* with *rml1* completely suppresses both the shoot and root growth [25]. Consistently, both root growth and shoot development are inhibited by the highly specific γ-ECS inhibitor, buthionine sulfoximine (BSO), in the *ntra ntrb* mutant, and the cytosolic TRXh3 is totally oxidized in the double knockout under this treatment, indicating that a GSH-dependent pathway takes charge of the alternative reduction of cytosolic TRXh3 [25]. A subgroup of the poplar h-type TRX isoforms is found to be reduced by GRXs [91]. Biochemical experiments demonstrate that GRXs, but not GSH, are able to reduce *Arabidopsis* TRXh3, suggesting that GRXs function as alternative reductants of TRXs in the *ntra ntrb* mutant [25]. Together, these results support an overlapping role for NTRs and GRXs in establishing the postembryonic activity in the apical meristems [25]. Genetic and biochemical characterization of the *ntra ntrb cad2* mutant further unravels that these two thiol reduction pathways interfere with plant developmental processes via modulating auxin signaling [26]. This triple mutant develops normally at the rosette stage, but later forms naked stems devoid of flower development. Besides, the *ntra ntrb cad2* plants show the lack of apical dominance, defects in vascular formation and reduced secondary root production [26]. All of these anomalous phenotypes are reminiscent of those transgenic and mutant plants impaired in auxin transport, signaling or biosynthesis [92,93,94,95,96,97,98]. Obviously, auxin levels and auxin transport capacities are significantly reduced in the *ntra ntrb cad2* mutant [26], highlighting a mechanistic link between thiol-based redox regulation and auxin signaling. In contrast with the *ntra ntrb rml1* mutant in which both the root and shoot apical meristematic activity is completely abrogated [25], the two apical meristems are not profoundly impaired in the *ntra ntrb cad2* mutant [26]. This phenotypic disparity conveys a threshold GSH concentration that is necessary for the development of postembryonic roots and shoots. Furthermore, the *ntra ntrb cad2* mutant defines a second threshold required for proper flower development, implying that flower development is more sensitive to redox perturbation than leaf development [26].

Mediator is an evolutionarily conserved transcriptional coregulator complex in all eukaryotes ranging from yeast to human. The plant Mediator is composed of 21 conserved and six plant-specific subunits [99]. Very recently, functional characterization of the *Arabidopsis* Mediator subunit 18 (MED18) has suggested a potentially synergistic role for GRXs and TRXs in plant immunity [100]. MED18 interacts with the putative repression domain of the zinc finger transcription factor YIN YANG1 (YY1), and YY1 is able to associate with the promoter regions of *GRXS13/ROXY18*, *GRXC9/ROXY19* and *TRXh5* [100]. Moreover, the *yy1* and *med18* mutants exhibit deregulated expression of* GRXS13/ROXY18*, *GRXC9/ROXY19* and *TRXh5*, which might contribute to their elevated susceptibility to necrotrophic fungal pathogens [100]. Accordingly, ectopic expression of *GRXS13/ROXY18* and *GRXC9/ROXY19* results in transgenic plants with a severe susceptibility to fungal infection [55,100]. Conversely, the *grxs13/roxy18* mutant shows increased resistance to *B. cinerea* [55]. In addition, the *trxh5* mutant is insensitive to the victorin toxin produced by the necrotrophic fungus, *Cochliobolus victoriae* [101]. These data together point to the direct transcriptional regulation of GRX and TRX genes by YY1, thereby preventing the expression of these disease susceptibility genes [100]. TRXs and GRXs are central players of the redox regulatory network, and virulence factors of necrotrophic fungi may interfere with the cellular redox equilibrium to promote disease susceptibility. Alternatively, pathogen-stimulated GRXs and TRXs may posttranslationally modify downstream signaling proteins, thus inactivating a specific resistance mechanism.

GRXs and TRXs function as major disulfide reduction enzymes that regulate the redox state of protein thiol groups. Biochemical and genetic studies demonstrate that these two systems share some common target proteins and operate in parallel in both developmental processes and defense responses. Elucidation of the functional redundancy between GRXs and TRXs, as well as dissection of the cross-communication between GRXs, TRXs and GSH are the key aspects of future studies on cellular redox signaling.

## 6. Conclusions and Perspectives

Over the last decade, our knowledge about the biological functions of GRXs in plants has drastically expanded (Table 2). Genetic dissection of available GRX mutants across the plant kingdom has enabled us to gain deep insight into their contribution to a plethora of plant processes [7,11,15,16,18,34,46,55,69,70,71]. An accumulating body of evidence indicates that GRX-mediated regulation of cellular redox homeostasis plays a potentially crucial role in posttranslational modifications of target proteins involved in organ development and defense responses against biotic and abiotic stresses in plants [25,46,47]. Given the ubiquity of the CPYC-type and CGFS-type GRXs to prokaryotes and eukaryotes, as well as the specificity of the CC-type GRXs to land plants, the involvement of GRXs in the assembly and delivery of iron-sulfur clusters seems to represent an ancestral GRX function. All three types of GRXs participate non-exclusively in oxidative stress responses caused by abiotic factors, revealing a conserved role for GRXs in scavenging ROS and maintaining the cellular redox balance. Strikingly, CC-type GRXs interact directly with TGA transcription factors to modulate two seemingly distinct biological processes,* i.e.*, floral organ development and plant immunity, suggesting a possible role for CC-type GRXs in posttranslationally modifying transcription factors and thereby affecting their DNA-binding and/or transcriptional activity. Only the number of CC-type GRXs increases during the evolution of land plants, underlining the notion that CC-type GRXs might have been recruited to develop complex plant architectures and adapt plants for constantly changing environments. Evidently, GRXs are involved in a wide variety of redox-controlled biological pathways, elucidating a key role for GRXs in modulating protein functions in a redox-dependent manner.

Monitoring physiological oxidants and measuring intracellular redox changes have recently been feasible due to the development of fluorescent redox biosensors, such as the redox-sensitive green fluorescent protein (roGFP), the redox-sensitive yellow fluorescent protein (rxYFP) and the GFP-based bioindicator, *HyPer*, specific for H_2_O_2_ [102,103,104]. A fascinating advantage of roGFP and HyPer over rxYFP is their ratiometric fluorogenic property. As genetically encoded redox probes, these biosensors can be targeted to specific subcellular compartments, enabling the global analysis of dynamic redox changes *in planta*. Oxidized roGFP can be reduced by the *Arabidopsis* CPYC-type GRXC1 [103], manifesting the potential of roGFP to monitor the intracellular redox homeostasis. Measuring the redox potential using roGFP reveals that the root cap and the meristematic zone have a more reduced redox status than the elongation zone in *Arabidopsis* roots [102], circumstantiating the key role for TRXs and GRXs in establishing the postembryonic root meristem [25,26]. The redox-sensing fluorescent probes can be engineered into model plants, offering powerful tools to dynamically image cellular redox processes.

**Table 2:** A list of functionally characterized GRXs from different plant species.

GRX	Type	Species	Function *
GRXC7/ROXY1 ^a^	CC	*Arabidopsis*	petal initiation and organogenesis, anther development and microspore formation [7,11,46]
GRXC8/ROXY2 ^a^	CC	*Arabidopsis*	anther development and microspore formation [7,46]
OsMIL1 ^a^	CC	rice	anther development and microspore formation [16]
MSCA1 ^a^	CC	maize	anther development and microspore formation [15]
GRXC11/ROXY4 ^b^	CC	*Arabidopsis*	GA-signaling and floral organ development [64]
GRXS13/ROXY18 ^a^	CC	*Arabidopsis*	disease susceptibility, photooxidative stresses [55,71,100]
GRXC9/ROXY19 ^b^	CC	*Arabidopsis*	Crosstalk between the SA and JA/ET defense pathways, disease susceptibility [12,60,100]
PvGRX5 ^c^	CGFS	fern	arsenic resistance, oxidative abiotic stresses [20,76,77]
SlGRX1 ^a^	CGFS	tomato	abiotic oxidative stresses [73]
GRXS17 ^a^	CGFS	*Arabidopsis*	temperature-dependent postembryonic growth and development, thermotolerance [70,72]
GRXS14 ^a^	CGFS	*Arabidopsis*	protection against protein oxidation [18]
GRXS14, 16 ^d^	CGFS	poplar	assembly and transfer of Fe-S clusters [17]
GRXS15 ^d^	CGFS	*Arabidopsis*	abiotic oxidative stresses [69]
GRXC1 ^a,d^	CPYC	*Arabidopsis*	assembly of Fe-S clusters, early steps after pollination [34]
GRXC2 ^a^	CPYC	*Arabidopsis*	early steps after pollination [34]
GRXC1 ^d^	CPYC	poplar	assembly of Fe-S clusters [8,19]

* The numbers within braces represent references; ^a–d^ The function of the GRX is determined by the phenotype of the corresponding null mutant ^a^, overexpression studies ^b^, ectopic expression in *E. coli* and *Arabidopsis* ^c^ or experiments performed* in vitro* and in yeast ^d^.

In spite of recent advances in deciphering plant GRXs, many gaps still remain in our understanding of their cellular functions, specific targets and biochemical activities. Photosynthetic organisms, particularly flowering plants, encompass a large number of GRXs [9]. However, only a few plant GRXs have been functionally characterized to date by means of genetic and biochemical approaches [7,11,15,16,18,34,46,55,69,70,71]. Genome-wide expression analysis suggests diverse roles of plant GRXs during various developmental stages, as well as in response to phytohormones, biotic and abiotic stresses [105,106]. Further functional analysis of plant GRXs and their potential targets will elucidate their contribution to different cellular processes. Apart from the three classical types of GRXs, the existence of GRX-like proteins in plants, such as the 4CxxC family, the CPFC/A class of the GRX/GST proteins, as well as the GRL-type GRXs, adds another layer of complexity to dissecting the GRX-mediated redox modulation of cellular processes [105,107]. Many attempts have been made to explore plant GRX targets via profiling the disulfide proteome and the S-glutathionylation proteome [78,108,109,110,111,112]. High-throughput proteomic techniques, including alkylation of free thiols followed by reduction of disulfide bonds and thiol affinity chromatography [110], incorporation of biotinylated GSH combined with two-dimensional polyacrylamide gel electrophoresis (2D-PAGE) and matrix-assisted laser desorption/ionization time of flight mass spectrometry (MALDI-TOF MS) [111], liquid chromatography with a mutated poplar GRX coupled to tandem MS [78], as well as cysteine labeling with a cysteine-reactive tandem mass tag (cysTMT) combined with tandem MS [112], allow for the large-scale identification of GRX targets implicated in all aspects of plant life processes. Up till now, combining proteomic approaches with protein interaction assays has uncovered a myriad of plant GRX targets involved in diverse cellular events [78,108,109,110,111,112]. Most of these target proteins are encoded by ubiquitously expressed housekeeping genes, and signaling proteins or transcription factors, which are generally encoded by spatiotemporally expressed genes, may escape these detection approaches. Then, *in planta* interactions of GRXs with their respective targets need further experimental confirmation. In particular, genetic experiments are required to explore whether GRXs act with their potential targets in the same cellular pathway. Despite a large array of putative GRX targets unraveled, it still remains a challenge to delineate the complete thiol-disulfide redox proteome modified by GRXs. Besides, the enzymatic properties of the CC-type GRXs have not yet been elucidated biochemically, though the CGFS-type GRXs possess the deglutathionylation activity and the CPYC-type GRXs display both the disulfide oxidoreductase and deglutathionylation activities [113,114]. As genetic experiments reveal that both the catalytic cysteine and the conserved glycine involved in GSH binding are indispensable for GRXC7/ROXY1 to exert its proper function, it has been postulated that the biochemical role of this type of GRXs is related to their oxidoreductase and deglutathionylation activities [11]. A second piece of experimental evidence in support of the CC-type GRXs having a catalytic activity arises from the artificially engineered CC-type GRXs, where the CP/GYC active site of the CPYC-type GRXs is replaced by the CCMC/S active site [37]. In contrast to the CPYC-type GRXs, all of the variants with the artificial CC-type active motif exhibit an impaired oxidoreductase activity, raising the possibility that the CCMC/S active sites of the genuine CC-type GRXs are permissive for the oxidoreductase activity, albeit being far less efficient than the CPYC-type GRXs. To substantiate that CC-type GRXs operate as oxidoreductases, it will be essential to produce soluble proteins corresponding to natural CC-type GRXs and further investigate their enzymatic activities.
